# Methodology for investigating moderating relationships in cognitive biases within workplace decision-making

**DOI:** 10.3389/fpsyg.2026.1705404

**Published:** 2026-05-08

**Authors:** Benjamin Ohms

**Affiliations:** Teesside University International Business School, Middlesbrough, United Kingdom

**Keywords:** behavioral economics, cognitive biases, decision avoidance, herding bias, moderation analysis, overconfidence bias, Singapore, workplace decision-making

## Abstract

While foundational theories of decision-making highlight the role of cognitive biases, there is limited understanding of how situational stressors dynamically alter these effects in organizational contexts. This study investigates how time pressure and task complexity moderate the influence of overconfidence, herding, and decision avoidance biases on critical stages of workplace decision-making. Utilizing data from 357 employees in Singapore, this study employs multiple linear regression with HC3 robust standard errors to test these complex interactions. The findings reveal that cognitive biases are not uniform; time pressure amplifies reliance on fast, heuristic processing, strengthening overconfidence and herding during information evaluation, while task complexity increases cognitive load, exacerbating decision avoidance. By explicitly detailing the mechanisms of these interactions, this study advances behavioral economics theory and provides a robust empirical framework for capturing the reality of non-uniform decision-making behavior under workplace stress.

## 1 Introduction

Decision-making is a fundamental aspect of human existence, heavily influenced by cognitive shortcuts known as heuristics and biases. Foundational theories by Herbert Simon, Daniel Kahneman, and Amos Tversky established that human rationality is bounded, leading individuals to rely on heuristics that can introduce systematic errors in judgment (Simon, 1955; Kahneman and Tversky, 1973, 1979; Dale, 2015; Gigerenzer, 2020). While these theories have been extensively applied to financial and investment contexts, there remains a critical conceptual gap regarding how these cognitive biases operate dynamically within general organizational decision-making (Ohms, 2025).

The critical theoretical gap in current behavioral economics literature lies in the static treatment of cognitive biases within organizational contexts. While foundational literature establishes the existence of biases such as overconfidence and herding, there is a distinct lack of dynamic, context-sensitive models that explain how these biases fluctuate under organizational stressors. This study addresses this conceptual void by positioning time pressure and task complexity not merely as environmental variables, but as primary moderating mechanisms that catalyze the shift between analytical (System 2) and heuristic (System 1) cognitive processing (Dale, 2015).

Furthermore, organizational decision-making is inherently context dependent. Direct relationships between cognitive biases and decision outcomes oversimplify the reality of the modern workplace, where external stressors interact with internal cognition (Phillips-Wren and Adya, 2020). To capture this complexity, it is theoretically necessary to extend beyond direct effects and employ moderation analysis. Specifically, this study introduces time pressure and task complexity as moderating mechanisms, recognizing them as significant stressors that alter cognitive processing and decision outcomes. By explicitly detailing the procedures for testing these complex interactions, this study contributes to behavioral economics theory by demonstrating how situational factors amplify or suppress cognitive biases, moving the field toward a more realistic and dynamic understanding of workplace behavior.

To capture the reality of modern workplaces, this study explicitly connects external stressors to Kahneman's System 1 and System 2 thinking. This study makes three key contributions: (1) advancing behavioral decision-making theory by demonstrating biases are not uniform under stress, (2) providing theoretically grounded mechanisms for this moderation, and (3) offering a robust empirical framework for testing these dynamics.

## 2 Theoretical foundations of moderation analysis

The evidence from prior systematic literature reviews consistently demonstrates that overconfidence bias, herding bias, anchoring bias, and loss aversion bias (closely related to decision avoidance bias) are the most prominent and frequently studied cognitive biases in decision-making. However, a limitation across nearly all these reviews is their focus on investment decision-making. There is a clear research gap highlighting the need to broaden the field of research to include other areas than investment. Furthermore, most studies lacked a broader focus on various industries and geographical locations (Shah et al., 2020; Rosyidah and Pratikto, 2022; Gatabi et al., 2023; Kunz and Sonnenholzner, 2023; Paul and Sundaram, 2023; Ahmad, 2024; Karki et al., 2024; Shah and Butt, 2024; Singh et al., 2024).

Furthermore, existing research on cognitive biases has predominantly treated biases as stable predictors of decision outcomes. However, this perspective overlooks a critical theoretical limitation: decision-making occurs under varying cognitive constraints, where situational stressors dynamically alter information processing. As a result, models that rely on direct effects fail to capture how biases fluctuate under conditions such as time pressure and complexity.

To understand how cognitive biases operate within organizational frameworks, it is necessary to ground the analysis in the concept of Bounded Rationality (Simon, 1955). Simon argued that the assumption of complete human rationality in rational choice theory is unrealistic (Simon, 1955; Gilovich et al., 2002; Tsoukiàs, 2008; Gigerenzer, 2020). He suggests that human decision-making is limited by cognitive understanding, available information, and time. When these limits are strained by situational stressors, individuals default to System 1 processing, a fast, automatic, and intuitive mode of thought that is highly susceptible to heuristics and biases (Dale, 2015). Conversely, System 2 processing is slower, analytical, and requires significant cognitive effort, which is often compromised in high-stress organizational environments (Dale, 2015). Time pressure and task complexity act as critical moderating forces that disrupt System 2 processing, compelling a heavier reliance on System 1 heuristics such as herding, overconfidence, and decision avoidance. Time pressure constrains cognitive resources, reducing analytical processing and increasing reliance on heuristics, while task complexity increases cognitive load, limiting processing capacity and promoting simplified or avoidant decision strategies.

Organizational decision-making is inherently context-dependent. Time pressure and task complexity are potent situational stressors that shift cognitive processing. Under time pressure, individuals are forced to reduce analytical processing (System 2) and increase their reliance on fast, heuristic-based thinking (System 1; Dale, 2015). This psychological mechanism suggests that time constraints amplify biases like overconfidence and herding to expedite decision-making. Conversely, task complexity directly strains Simon's bounded rationality by increasing cognitive load. When decisions lack clear solutions and overwhelm cognitive capacity, individuals succumb to decision avoidance or rely on simplified rules. Understanding these psychological mechanisms requires explicitly testing them as moderators, as the direct relationship between cognitive biases and decision outcomes is not uniform across different environmental stressors.

Understanding moderating effects is fundamental in behavioral economics and management research, especially when investigating complex human behaviors in organizational contexts. Direct effects alone may oversimplify the reality of decision-making, where various internal and external factors interact (Phillips-Wren and Adya, 2020). By exploring how time pressure and complexity moderate the influence of biases such as overconfidence, herding, and decision avoidance, this study can generate insights for improving decision quality in the Singaporean workplace. Specifically, this study conceptualizes cognitive biases as context-sensitive processes rather than stable traits, demonstrating how their effects systematically vary under environmental stressors.

## 3 Conceptual framework and grouped hypotheses

The research framework and hypothesis can be divided into three sections. Direct effect, moderating effect with time pressure and moderating effect with complexity.

### 3.1 Direct effects of cognitive biases

**Figure 1:**
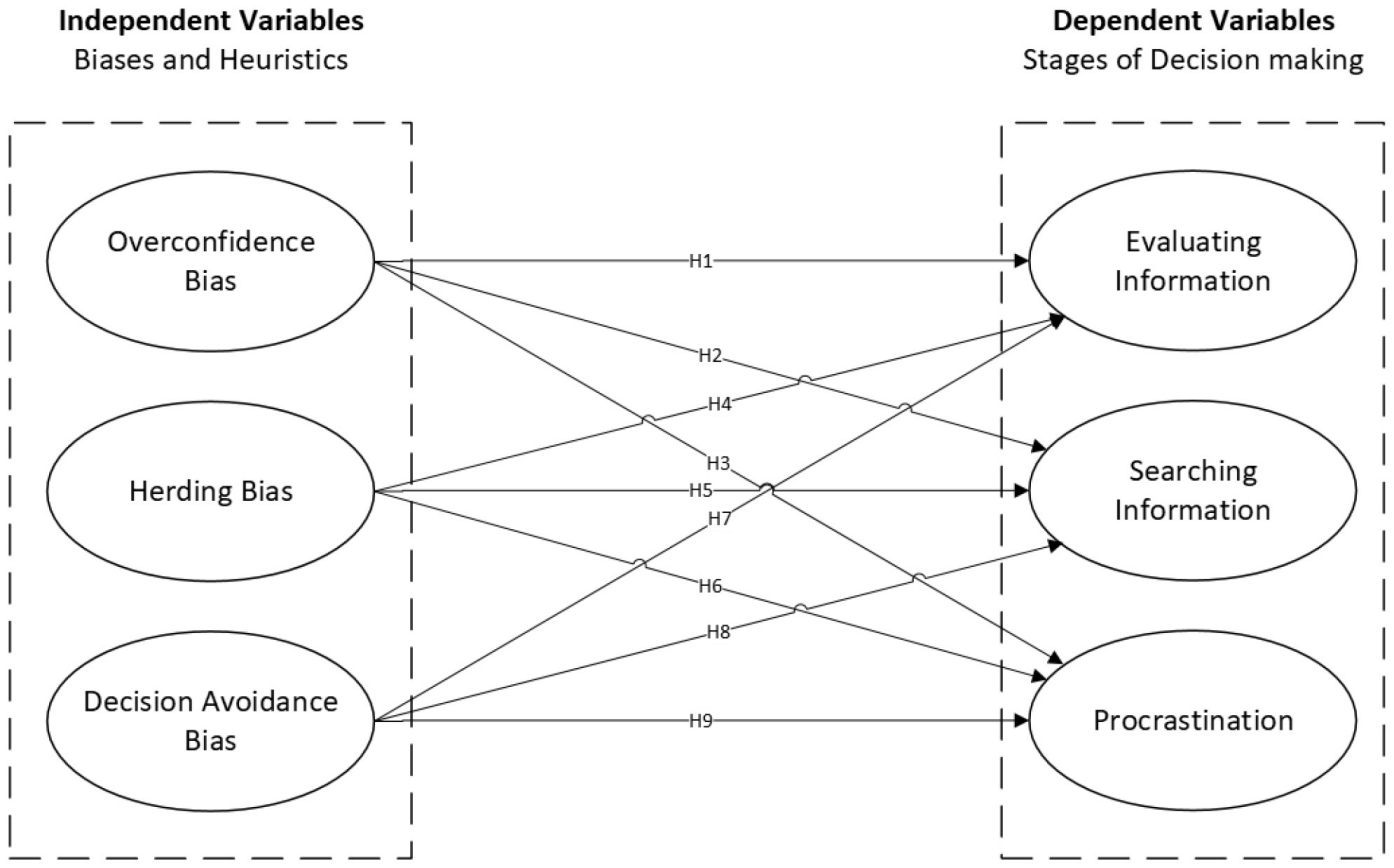
Research framework—base model.

Overconfidence, herding, and decision avoidance directly interfere with objective rationality by skewing how information is sourced and judged. We anticipate these biases will show baseline relationships with how employees search for information, evaluate alternatives, and procrastinate (Figure 1).

*H1–H9: Overconfidence, herding, and decision avoidance biases have significant direct relationships with evaluating information, searching information, and procrastination*.

### 3.2 Moderating effects of time pressure

**Figure 2:**
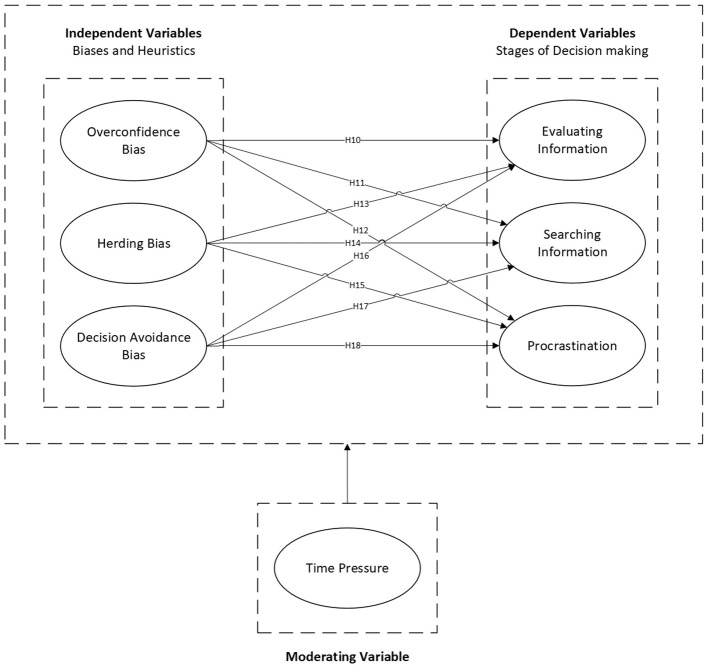
Research framework—extended model 1.

Time pressure reduces analytical capacity, forcing a shift to System 1 heuristic processing. Because urgency demands immediate action, we anticipate it will strengthen reliance on fast-acting biases (overconfidence, herding) while suppressing inaction (procrastination). Refer to Figure 2.

*H10–H18: Time pressure significantly moderates the relationships between cognitive biases and decision-making stages, specifically by amplifying the effects of overconfidence and herding on information evaluation/search, while weakening the effect of biases on procrastination*.

### 3.3 Moderating effects of complexity

**Figure 3:**
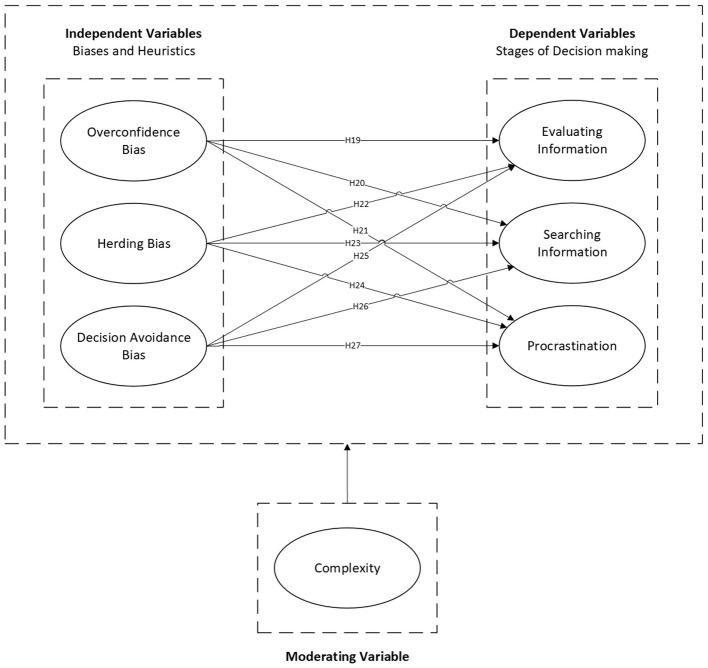
Research framework—extended model 2.

Task complexity increases cognitive load and obscures clear solutions, heavily taxing bounded rationality. This overwhelming of cognitive resources forces reliance on simplified decision rules or complete task avoidance. Refer to Figure 3.

*H19–H27: Task complexity significantly moderates the relationships between cognitive biases and decision-making stages, specifically by strengthening the effects of decision avoidance and overconfidence on procrastination as cognitive load increases*.

## 4 Data characteristics and prior validation

### 4.1 Data collection and sample

This study utilized a quantitative cross-sectional survey design, targeting employees across all hierarchical levels and industry sectors within Singapore. Data were collected in April 2025 using an online questionnaire comprising closed-ended, five-point Likert-type scale questions adapted from established scales. From an initial invitation to 2,861 individuals, 357 valid responses were successfully collected, exceeding recommended sample size thresholds for robust regression modeling and representing a broad view of workplace biases free from specific industry constraints (Saunders, 2009; Kyriazos, 2018; Kline, 2023).

### 4.2 Data management and validation

A preliminary check confirmed no missing data. Univariate outliers were excluded using *z*-scores (>3.29), and multivariate outliers were managed using the Mahalanobis distance (*p* < 0.001). While normality assessments (Shapiro–Wilk and Kolmogorov–Smirnov) indicated non-normal distributions, the sample size of 357, coupled with the use of robust standard errors and the use of Likert scale data, safely allows for the application of parametric regression testing in accordance with the Central Limit Theorem (Casson and Farmer, 2014; Kwak and Kim, 2017). As large sample sizes combined with the use of HC3 robust standard errors effectively mitigate non-normality violations in OLS regression without compromising coefficient consistency (Long and Ervin, 2000; Hayes and Cai, 2007). Moreover, (DeWees et al. 2020) argued that parametric methods can also be used for non-normally distributed data. They specifically mentioned that Likert-scale data is robust enough to allow the use of parametric methods even under non-normal distribution and with small sample sizes.

The reliability of the measurement instruments was confirmed using Cronbach's Alpha, with variables demonstrating satisfactory internal consistency ranging from 0.679 to 0.940. Although Searching Information (SI) showed a slightly lower value of 0.679, this was deemed acceptable due to its fewer measuring items (three items) and satisfactory item-total correlations (all above 0.3; Tavakol and Dennick, 2011; Yip et al., 2012).

## 5 Methodological procedures for moderation analysis

To test the interaction effects (H10–H27), multiple linear regression was utilized. To mitigate multicollinearity inherent in interaction terms, all independent variables (OB, HB, and AB) and moderating variables (TP, CO) were strictly mean-centered prior to the construction of product terms. Six interaction terms were computed (e.g., Overconfidence Bias × Time Pressure).

Given the inclusion of multiple interaction terms, multicollinearity was carefully monitored. Variance Inflation Factor (VIF) diagnostics confirmed that collinearity remained well-below the conservative threshold of five, ensuring stable coefficient estimation.

A critical consideration identified during preliminary analysis was the presence of heteroscedasticity. To address this and ensure robust statistical inference, the regression models were estimated using HC3 (Heteroscedasticity-Consistent Covariance Matrix Estimator, type 3) robust standard errors. HC3 is methodologically superior for producing accurate standard errors and p-values in heteroscedastic datasets, particularly for samples under *N* = 500. According to (Long and Ervin 2000); (Hayes and Cai, 2007), HC3 (Heteroscedasticity-Consistent Covariance Matrix Estimator, type 3) is a robust estimator that yields more accurate standard errors and *p*-values in heteroscedasticity. HC3 estimators are strictly recommended for maintaining statistical power and correcting for heteroscedasticity, outperforming HC0–HC2 in finite samples, making it the most rigorous choice for cross-sectional behavioral data (Long and Ervin, 2000; Hayes and Cai, 2007).

The interaction terms directly correspond to hypotheses H10–H27, allowing for empirical testing of the proposed moderating effects of time pressure and task complexity on the relationships between cognitive biases and decision-making outcomes.

## 6 Ethical considerations

Adherence to strict ethical guidelines was maintained. The approval was obtained from the Teesside University Ethics Committee before data collection. Participant anonymity was guaranteed, with no personally identifiable information collected. Comprehensive Participant Information Sheets were provided to all participants. Informed consent was obtained via Participant Consent Forms. These measures ensure that the research is conducted with the highest ethical standards, protecting participant welfare and data privacy.

## 7 Conclusion

This study provides a theoretically grounded and statistically robust methodology for evaluating how cognitive biases function within organizational decision-making under stress. By advancing beyond direct-effect models, this approach introduces time pressure and task complexity as critical moderating mechanisms that dynamically alter cognitive processing in the workplace.

Theoretically, this framework extends the application of bounded rationality out of the financial sector and into general organizational behavior. It provides a blueprint for understanding how high-stress, complex environments force reliance on fast, heuristic thinking and amplify biases like overconfidence and decision avoidance. Practically, the analytical procedures outlined herein, specifically the application of HC3 robust standard errors and mean-centered interaction terms, enable researchers to accurately capture these nuanced, non-uniform relationships. Organizations can use these insights to identify exactly under which environmental conditions decision quality deteriorates, paving the way for targeted structural interventions.

## 8 Limitations and future directions

While this study provides robust empirical evidence for the moderating roles of time pressure and complexity, the cross-sectional design inherently limits causal inference. Cross-sectional survey data captures a specific moment in time, meaning potential endogeneity cannot be entirely ruled out. Future research should employ longitudinal designs or experimental methodologies with instrumental variables to further isolate the causal mechanisms by which these stressors amplify or suppress cognitive biases over time.

## Data Availability

The data for this study, comprising the survey responses, are openly available on the Open Science Framework at https://doi.org/10.17605/OSF.IO/5B7QM.
